# Which Factors Affect Functional Outcomes and Survival in Metastatic Humeral Fractures Treated With Nails?

**DOI:** 10.1111/os.70101

**Published:** 2025-06-29

**Authors:** Ebubekir Eravsar, Ali Gulec, Sadettin Ciftci, Numan Mercan, Selim Safali, Bahattin Kerem Aydin

**Affiliations:** ^1^ Department of Orthopedics and Traumatology Selcuk University Faculty of Medicine Konya Turkiye; ^2^ Department of Orthopedics and Traumatology Kahramanmaraş Necip Fazıl City Hospital Kahramanmaraş Turkiye

**Keywords:** cement, humerus, metastasis, nail, pathological fracture, survival

## Abstract

**Objective:**

Intramedullary nailing is a treatment method for metastatic humerus fractures that stabilizes a large area while minimizing damage to the surrounding soft tissues. However, the results of this treatment may vary depending on certain factors. This study aimed to investigate the factors influencing functional outcomes and survival in patients with pathological humeral fractures treated using humeral nails.

**Methods:**

This retrospective study included 41 patients who underwent humeral nailing for metastatic pathological humerus fractures between 2009 and 2024. Functional outcomes were compared based on factors such as gender, age, cancer type, another pathological fracture surgery, visceral metastases, cancer diagnosis prior to fracture, fracture type and location, and cement use, using VAS improvement, MSTS, KPS scores, and ROM measurement. Survival analysis was performed considering these same factors. Statistical analyses included the Mann–Whitney *U* test, Kruskal‐Wallis test, Chi‐square test, and Kaplan–Meier survival curves. Cox regression analyses were used to identify factors associated with mortality.

**Results:**

In younger patients, better VAS improvement(*p* = 0.001), MSTS(*p* = 0.038), KPS(*p* = 0.028), and ROM(*p* = 0.045) were observed compared to those 65 and older. Cancer type and visceral metastases negatively impacted MSTS(*p* = 0.007, *p* = 0.049) and KPS(*p* = 0.002, *p* = 0.022). Actual fractures showed greater VAS improvement than impending fractures(*p* = 0.002), and shaft fractures had greater VAS improvement than proximal fractures(*p* = 0.037). Unknown cancer diagnosis prior to fracture led to better VAS improvement(*p* = 0.008), MSTS(*p* = 0.018), KPS(*p* = 0.023), and ROM(*p* = 0.006). Rapid growth tumor(*p* < 0.001) and visceral metastasis(*p* = 0.007) were independently associated with poor survival. No significant effects were seen for gender or cement use on functional outcomes and mortality.

**Conclusion:**

Although intramedullary nails are feasible implants for humeral pathological fractures, there are significant factors that affect their functional outcomes and survival. Actual fractures and shaft fractures showed better pain relief. Patients with a known cancer diagnosis prior to fracture and older patients had poor functional outcomes. Rapid cancer type and visceral metastasis negatively affect both functional outcomes and survival. Although cement use carries a risk of thrombosis, no significant changes in mortality and functional outcomes were observed with cement use.

**Level of Evidence:**

IV.

## Graphical abstract

This study examines factors affecting functional outcomes and survival in 41 patients with metastatic humeral fractures treated with intramedullary nails. Actual fractures and shaft fractures showed better pain improvement. Patients with a known cancer diagnosis prior to fracture and older patients had poor functional outcomes. Rapid cancer type and visceral metastasis negatively affect both functional outcomes and survival. Cement use had no significant impact on mortality or functional outcomes.

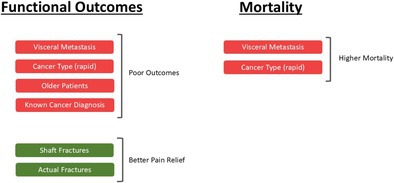

## 1 Introduction

The humerus is the second most common long bone to undergo metastasis [1, 2, 3]. Metastases can lead to structural changes in the bone, which may manifest at the cellular level as osteolysis, osteopenia, osteoblastic activity, or combinations thereof. Among these, lytic and destructive lesions are the most frequently observed [4]. These lesions may result in actual or impending pathological fractures, which significantly impair functional capacity in cancer patients. Consequently, surgical treatments play a predominant role in the management of such pathological fractures.

As cancer treatments improve, patients' life expectancy is extending. However, this progress has also led to an increase in issues like metastatic bone disease and the related pathological fractures. Fractures caused by metastatic bone lesions have limited healing potential. Therefore, the primary goals of surgical treatments are to restore the patient's functionality and alleviate pain [5, 6]. Surgical options for the treatment of humeral pathological fractures include intramedullary nails [5, 7, 8, 9, 10], plate‐screw systems [11] and endoprostheses [12]. The choice of surgical treatment is determined by factors such as the location of the lesion, bone quality, life expectancy, the patient's overall condition, and the surgeon's preference [13, 14]. Achieving successful stabilization with minimal operative morbidity is crucial for these patients [15]. Intramedullary nails stand out among these options due to their biomechanical advantages and ability to provide whole humerus protection, in addition to the aforementioned characteristics. However, the functional outcomes and mortality rates of patients undergoing this procedure can vary considerably among cancer patients. In fact, previous studies have reported that the one‐year survival rate of patients with humeral metastatic lesions ranges from 15% to 60% [14, 16, 17, 18]. Additionally, when considering the variations in the functional scores observed in earlier research [4, 8, 9, 19, 20], it is reasonable to hypothesize that various factors may contribute to these differing outcomes. These factors may include patient and surgery related variables such as age, cancer type, the presence of visceral metastases, cement use, and fracture characteristics, all of which could play a crucial role in influencing both survival and functional recovery.

In this study, patients with pathological fractures of the humerus caused by metastasis were examined. The primary aim of this study is to investigate the effects of factors such as gender, age, cancer type, another pathological fracture surgery, visceral metastases, cancer diagnosis prior to fracture, fracture type and location, and cement use on functional outcomes in patients with pathological humeral fractures treated with humeral nails. The secondary aim of this study is to investigate whether these factors have an impact on mortality.

## 2 Methods

### 2.1 Study Design and Setting

This study was a retrospective study. Ethical approval was obtained from the local ethics committee (Selcuk University Local Ethics Committee, Date: 03.12.2024, Number: 2024/604). The data were retrieved from the hospital's electronic archives and the clinical archives, where detailed manual recordings of patient measurements were maintained. The study was conducted in the orthopedics department of a university hospital. Within this hospital, there is a multidisciplinary orthopedic oncology council, and treatment decisions are made by this council.

### 2.2 Patients

Between 2009 and 2024, the records of 66 patients who developed pathological fractures of the humerus due to metastatic disease and were indicated for surgical treatment by the orthopedic oncology council were reviewed. The inclusion criteria for this retrospective study were: age ≥ 18 years, presence of an actual or impending pathological fracture of the humerus due to metastatic disease, treatment with intramedullary nailing, and availability of both preoperative and postoperative clinical data in hospital records. Exclusion criteria included: refusal of surgical treatment (*n* = 3), treatment with plate‐screw fixation or endoprosthetic replacement instead of intramedullary nailing during initial fracture management (*n* = 18), and loss to follow‐up due to continuation of oncological treatment at another institution (*n* = 4). Consequently, data from 41 patients who met the eligibility criteria were included in the final analysis.

### 2.3 Descriptive Data

Among the 41 patients included in the study, 15 were female and 26 were male. The mean age of the patients was 63.9 ± 10.2 years. The average follow‐up period was found to be 10.1 ± 10.9 months. The primary cancer diagnoses were as follows: breast cancer in 10 patients, multiple myeloma in 9 patients, lung cancer in 8 patients, renal cell carcinoma in 5 patients, prostate cancer in 4 patients, thyroid cancer in 2 patients, bladder cancer in 1 patient, hepatocellular cancer in 1 patient, and colon cancer in 1 patient. At the time of fracture, 34 patients had a known cancer diagnosis, while 7 were diagnosed after the fracture. In patients without a cancer diagnosis prior to the fracture, comprehensive diagnostic investigations were conducted before surgery to verify that the lesion represented a metastasis from an underlying malignancy rather than a primary bone tumor. Additionally, 38 patients had multiple bone metastases, and 11 patients had a history of another surgeries due to pathological fractures in other bones. Visceral metastases were present in 16 patients. Regarding the location of fractures, 15 pathological fractures were in the proximal humerus, and 26 were in the humeral shaft. None of the fractures extended to the distal or proximal joints. In terms of fracture type, 27 fractures were classified as actual pathological fractures, while 14 were impending pathological fractures.

### 2.4 Treatment Plan, Surgery, and Follow‐Up

All patients had their treatment plans discussed preoperatively in an orthopedic oncology council. Surgery was performed under general anesthesia for all patients. The mean operative time for all patients was 74.3 ± 19.7 min. During the surgical procedure, curettage and cementing were applied to the fracture site in 24 patients, while 17 patients underwent nailing without cement. The use of cement had no specific indication and was based on the surgeon's intraoperative preference. One patient died intraoperatively during the cementing process. A total of five patients died within the first 2 weeks postoperatively. Postoperative oncological treatments (radiotherapy/chemotherapy) were administered by the orthopedic oncology council within 2–3 weeks after surgery. Except for the five patients who experienced early mortality, all patients received postoperative oncological treatments. Prolonged wound drainage was observed in four patients, which resolved with dressing changes without the need for additional surgical intervention. Revision surgery with endoprosthesis was performed in one patient due to rapid progression of the tumoral lesion in the humerus. Cement extrusion into soft tissues was noted in eight patients who underwent cementing; however, no permanent neurovascular injuries were observed in the study cohort. Radial nerve neuropraxia occurred in one patient who underwent nailing without cement, and it resolved spontaneously. The average time from fracture to surgery was 12.0 ± 4.1 days. In patients without a prior cancer diagnosis, the average time from fracture to surgery was 16.7 ± 3.5 days, and in patients with an established diagnosis, it was 11.1 ± 3.6 days. As of December 2024, the number of surviving patients was six. Postoperatively, patients were encouraged to engage in early mobilization. Follow‐up evaluations were conducted biweekly during the first month after surgery, followed by monthly assessments throughout the first year. For patients surviving beyond 1 year, follow‐ups were scheduled every 3 months.

### 2.5 Evaluations

Functional outcomes were assessed using Visual Analog Scale (VAS) improvement, Musculoskeletal Tumor Society (MSTS) score, Karnofsky Performance Status (KPS) score, and range of motion (ROM) measurements. Pain was assessed using the VAS scale during both preoperative and postoperative periods, where patients rated their pain on a scale from 0 to 10, with higher scores reflecting greater pain severity. The difference between the patients' preoperative and postoperative VAS scores was referred to as “VAS improvement.” The MSTS score ranges from 0 to 30, with higher scores indicating better functionality, while the KPS score ranges from 0 to 100, with higher scores representing better performance. The shoulder ROM on the affected side was calculated as a percentage compared to the opposite side. The VAS improvement, MSTS scores, KPS scores, and ROM percentages of the patients were evaluated according to gender, age, type of cancer, history of another pathological fracture surgery, presence of visceral metastases, cancer diagnosis prior to fracture, fracture type, fracture location, and cement use. The type of cancer was categorized into slow, moderate, and rapid‐growing groups according to the classification by Katagiri et al. [21]. In the age‐related analysis, patients were categorized into two groups: those under 65 and those aged 65 years or older, based on the World Health Organization's classification of individuals aged 65 and above as “elderly.” Since the evaluations of the 5 patients who died within the first 2 weeks could not be performed, the aforementioned analyses were conducted for 36 patients.

Survival analysis was conducted on 41 patients. The analysis was based on gender, age, type of cancer, history of another pathological fracture surgery, presence of visceral metastases, cancer diagnosis prior to the fracture, fracture type, fracture location, and cement use.

### 2.6 Statistical Analysis

Assumptions of normality were checked using the Kolmogorov–Smirnov and Levene's tests. Nonparametric tests were used when these assumptions were violated or for ordinal data. Group comparisons were made using the Mann–Whitney *U* test for comparisons between two groups and the Kruskal–Wallis test for comparisons among more than two groups. The Chi‐square test was used to investigate associations between categorical variables. Significance levels were set at 0.05. Survival curves were estimated using the Kaplan–Meier method. The impact of risk factors on mortality was assessed using univariate and multivariate Cox regression analyses. In univariate analyses, a significance level of 0.1 was used, while in multivariate analyses, the significance level was set at 0.05. The Backward‐Wald method was used for variable selection in the multivariate analysis. Results were presented as Hazard Ratios with 95% Confidence Intervals. All statistical analyses were performed using SPSS 22 software (IBM, Armonk, NY, USA).

## 3 Results

### 3.1 Functional Outcomes

The mean VAS improvement of the patients was found to be 5.8 ± 1.7. Postoperative MSTS scores were 19.7 ± 2.8, and KPS scores were 65.0 ± 10.8. The shoulder ROM compared to the opposite side was found to be 77.2 ± 17.0. In patients under 65 years of age, better results were observed in terms of VAS improvement (*p* = 0.001), MSTS (*p* = 0.038), KPS (*p* = 0.028), and ROM (*p* = 0.045) compared to patients aged 65 and above. Type of cancer and presence of visceral metastases had a negative impact on MSTS (*p* = 0.007 and *p* = 0.049) and KPS (*p* = 0.002 and *p* = 0.022). Patients who had undergone surgery for another pathological fracture had lower ROM scores (*p* = 0.027). In actual pathological fractures, greater VAS improvement was observed compared to impending fractures (*p* = 0.002), and in shaft fractures compared to proximal fractures (*p* = 0.037). Patients with an unknown cancer diagnosis prior to the fracture had better VAS improvement (*p* = 0.008), MSTS (*p* = 0.018), KPS (*p* = 0.023), and ROM (*p* = 0.006) scores compared to those with a known diagnosis. No significant effects of gender or cement use were observed on functional outcomes (Table 1). Patients treated with intramedullary nailing without cement had a mean operative time of 57.9 ± 16.0 min, while those with cement application had a significantly longer operative time of 85.9 ± 12.7 min (*p* < 0.001). There was no association between cement use and fracture type (*p* = 0.228). Similarly, no significant relationship was found between cement use and the type of cancer (*p* = 0.832).

**TABLE 1:** Pain, functional and ROM outcomes of patients.

	VAS improvement		MSTS		KPS		ROM	
	Median (IQR)	*p*	Median (IQR)	*p*	Median (IQR)	*p*	Median (IQR)	*p*
Gender								
Female	6 (3)	0.845	20 (4)	0.328	70 (15)	0.131	80 (25)	0.379
Male	6 (3)		20 (6)		70 (20)		70 (40)	
Age								
Below 65	7 (3)	0.001*	20 (2)	0.038*	70 (0)	0.028*	80 (30)	0.045*
65 and above	5 (2)		18 (5)		60 (20)		70 (25)	
Type of cancer								
Slow	6 (3)	0.335	22 (2)	0.007*	70 (10)	0.002*	90 (30)	0.052
Moderate	6 (3)		18 (3)		60 (10)		70 (15)	
Rapid	5 (3)		18 (4)		50 (20)		60 (30)	
Another pathological fracture surgery								
No	6 (3)	0.083	20 (4)	0.059	70 (10)	0.077	80 (30)	0.027*
Yes	5 (2)		18 (4)		60 (20)		65 (28)	
Visceral metastasis								
No	6 (4)	0.631	20 (4)	0.049*	70 (10)	0.022*	80 (30)	0.084
Yes	6 (3)		18 (4)		60 (20)		70 (20)	
Cancer diagnosis prior to fracture								
No	8 (1)	0.008*	22 (2)	0.018*	70 (10)	0.023*	100 (20)	0.006*
Yes	5 (3)		18 (4)		60 (10)		70 (25)	
Fracture type								
Impending	4.5 (2)	0.002*	20 (3)	0.343	70 (3)	0.160	70 (13)	0.737
Actual	7 (2)		18 (4)		60 (13)		80 (40)	
Fracture location								
Proximal	4 (3)	0.037*	20 (3)	0.697	70 (5)	0.361	80 (20)	0.697
Shaft	6 (2)		18 (4)		60 (10)		70 (40)	
Cement								
No	6 (2)	0.133	20 (4)	0.975	70 (20)	0.924	80 (40)	0.612
Yes	5 (3)		20 (4)		70 (10)		70 (15)	

Abbreviations: KPS: Karnofsky Performance Scale; MSTS: Musculoskeletal Tumor Society Score; ROM: range of motion; VAS: Visual Analog Scale.

* *p* values were less than 0.05.

### 3.2 Mortality Outcomes

**FIGURE 1:**
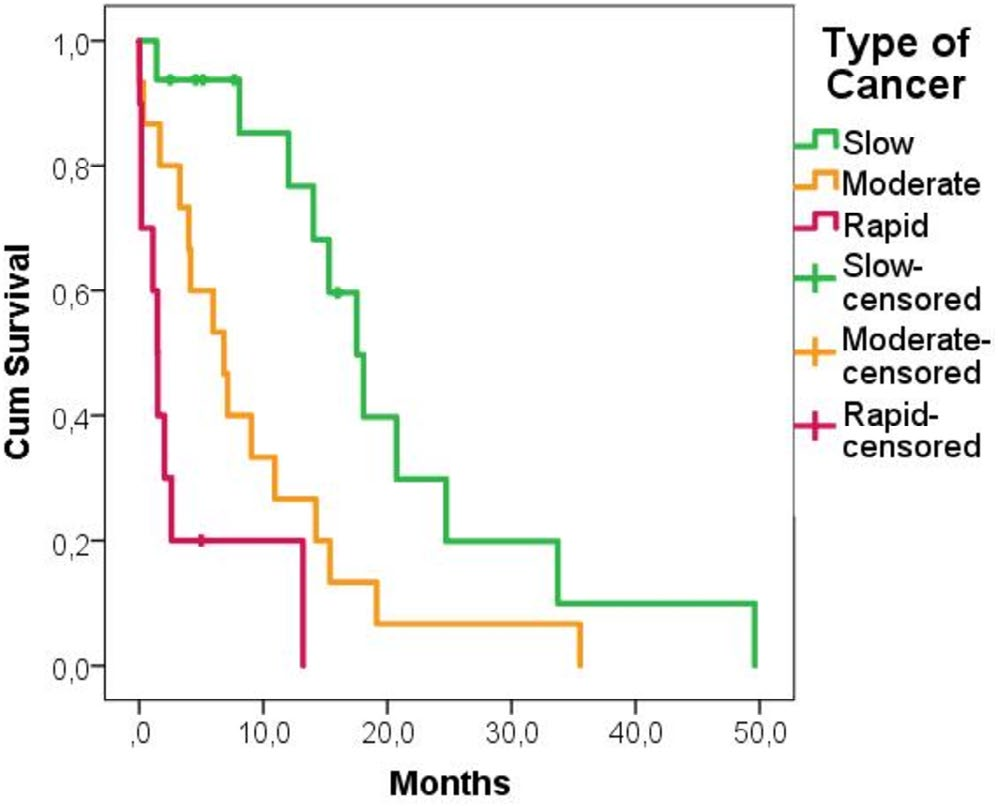
Kaplan–Meier survival curves showing the impact of type of cancer.

**FIGURE 2:**
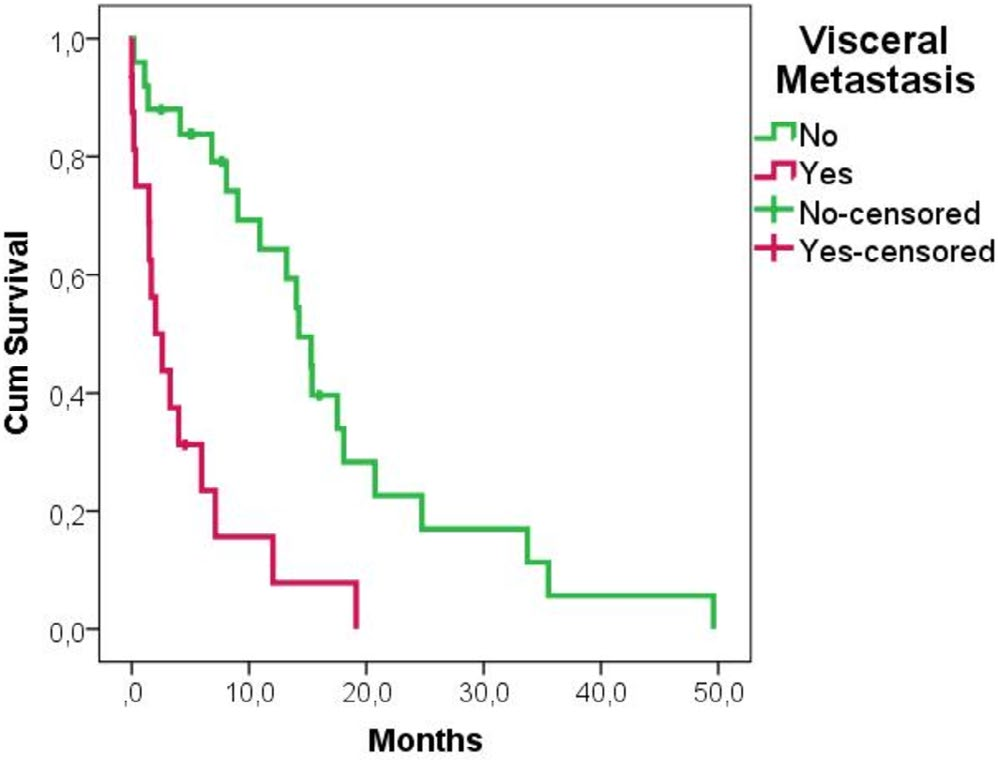
Kaplan–Meier survival curves showing the impact of visceral metastasis.

The cumulative 1‐year survival rate was 37%. According to the univariate analysis, four factors were deemed suitable for further analysis. Type of cancer (moderate: *p* = 0.017 and rapid: *p* < 0.001), another pathological fracture surgery (*p* = 0.028), visceral metastasis (*p* < 0.001) and cancer diagnosis prior to fracture (*p* = 0.067) were found to be statistically significant in univariate analysis. Other potential factors including gender, age, fracture type, fracture location, and cement usage were not associated with mortality (Table 2). In multivariate analysis, rapid growth tumor (HR = 6.963 [95% CI, 2.364–20.510], *p* < 0.001) (Figure 1) and visceral metastasis (HR = 3.098 [95% CI 1.360–7.060] *p* = 0.007) (Figure 2) were independently associated with poor survival (Table 3 **)**.

**TABLE 2:** Univariate Cox regression analysis.

Factors		Hazard ratio (%95 CI)	*p*
Gender	Female (*n* = 15)	Reference	
	Male (*n* = 26)	0.707 (0.349–1.432)	0.336
Age	Below 65 (*n* = 20)	Reference	
	65 and older (*n* = 21)	1.351 (0.685–2.665)	0.386
Type of cancer	Slow (*n* = 16)	Reference	
	Moderate (*n* = 15)	2.679 (1.189–6.037)	0.017*
	Rapid (*n* = 10)	8.665 (3.099–24.225)	< 0.001*
Another pathological fracture surgery	No (*n* = 30)	Reference	
	Yes (*n* = 11)	2.581 (1.108–6.012)	0.028*
Visceral metastasis	No (*n* = 25)	Reference	
	Yes (*n* = 16)	4.052 (1.903–8.628)	< 0.001*
Cancer diagnosis prior to fracture	No (*n* = 7)	Reference	
	Yes (*n* = 34)	2.454 (0.940–6.411)	0.067*
Fracture type	Impending (*n* = 14)	Reference	
	Actual (*n* = 27)	1.748 (0.854–3.577)	0.127
Fracture location	Proximal (*n* = 15)	Reference	
	Shaft (*n* = 26)	1.305 (0.639–2.663)	0.465
Cement	No (*n* = 17)	Reference	
	Yes (*n* = 24)	1.137 (0.574–2.251)	0.713

Abbreviation: CI: confidence interval.

* *p* values were less than 0.1.

**TABLE 3:** Multivariate cox regression analysis.

Factors		Hazard ratio (%95 CI)	*p*
Type of cancer	Slow	Reference	
	Moderate	1.913 (0.805–4.545)	0.142
	Rapid	6.963 (2.364–20.510)	< 0.001*
Visceral metastasis	No	Reference	
	Yes	3.098 (1.360–7.060)	0.007*

* *p* values were less than 0.05.

## 4 Discussion

In this study, patients with pathological fractures of the humerus caused by metastasis were investigated. The primary aim was to evaluate the impact of various factors including gender, age, cancer type, another pathological fracture surgery, visceral metastases, cancer diagnosis prior to fracture, fracture type and location, and cement use on functional outcomes following humeral nailing. The secondary aim was to assess whether these factors also influence patient mortality. This study found that cancer type and the presence of visceral metastasis negatively influenced functional outcomes. Younger patients and patients without a prior cancer diagnosis before the fracture had better functional scores. Greater pain relief was observed in actual fractures compared to impending fractures, and in shaft fractures compared to proximal fractures. Regarding survival, rapid tumor type and visceral metastasis were independently associated with higher mortality, while no other factors showed a significant impact. Although cement use carries intraoperative and postoperative risks, it did not significantly affect either mortality or functional outcomes.

### 4.1 Functional Outcomes and Associated Factors

Previous studies have shown that in metastatic pathological fractures, humeral nailing results in a VAS improvement of 6–7 units for pain relief, and postoperative KPS scores range from 75 to 80 [9, 19]. Studies examining MSTS scores have found that postoperative scores range from an average of 19 to 28 [4, 8, 9, 19, 20]. In evaluations of ROM, there are studies that report the majority of patients did not achieve full ROM [20], as well as studies indicating that good ROM values were achieved in a sufficient number of patients [19]. In this study, the mean VAS improvement of the patients was found to be 5.8 ± 1.7, the postoperative MSTS scores 19.7 ± 2.8, and the KPS scores were 65.0 ± 10.8. Differing from other studies, several factors believed to affect functional outcomes were compared, yielding noteworthy results. For instance, worse outcomes were observed in older patients, and it was found that the type of cancer and the presence of visceral metastasis negatively affected functional scores. Thus, these findings can be interpreted as the natural consequences of age and disease progression [22]. Pain resulting from actual fractures may be more severe than pain caused by impending fractures. Therefore, it is quite reasonable to expect greater pain relief in patients with actual fractures. The greater pain relief observed in the shaft region compared to the proximal region is a phenomenon whose mechanism is not fully understood. However, this could be interpreted as fractures in the middle area of the bone being more prone to displacement and being managed with U‐slabs during the preoperative period, which may result in more pain compared to proximal humerus fractures. For this reason, VAS improvement may be more significant in shaft fracture patients. Patients who did not have a cancer diagnosis prior to the fracture had better outcomes compared to those with a prior diagnosis. This may be due to the fact that patients who were undiagnosed until the fracture had less severe cancer‐related symptoms. Therefore, more successful results may be achieved in patients with better general condition and fewer complaints.

### 4.2 Mortality Outcomes and Associated Factors

Studies examining factors affecting the survival of patients with pathological humeral fractures are very limited in the literature, and there are only a few studies on this topic. In the study by Wedin et al., lung cancer, poor and moderate KPS scores, visceral metastases, and more than three skeletal metastases were found to be associated with lower survival [14]. According to the multivariate analysis by Wisanuyotin et al., the type of primary cancer and ECOG performance status were found to be associated with mortality. However, these analyses showed that visceral metastasis, the onset of metastatic bone disease, and the presence of pathological fractures did not affect mortality [17]. Bayram et al. found in their multivariate analysis that rapid growth tumors, the presence of pathological fractures in other extremities, and ECOG performance score were independently associated with worse overall survival. However, they also found in this analysis that visceral metastasis and the onset of metastatic bone disease were not associated with worse survival [18]. In the other studies, the survival rate of patients with humeral metastatic lesions varies between 15% and 60% for 1 year [14, 16, 17, 18]. In our study, the 1‐year survival rate of the patients was found to be 37%. Additionally, while our study's results overlap with some of the findings in the mentioned studies, there are also discrepancies. Specifically, in this study, the multivariate analysis showed that the rapid tumor type and visceral metastasis independently increased the risk of death; whereas, in the univariate analysis, fracture type did not affect survival, and in the multivariate analysis, another pathological fracture surgery and cancer diagnosis prior to fracture surgery were also found not to affect survival.

One of the notable factors compared in the study is the use of cement. There are differing opinions regarding the intramedullary nailing technique, and no consensus has been reached on whether or not cement should be used [15, 19, 20]. The main purpose of cementing is to enhance fixation and fill the void, especially in fractures with large defects [23]. However, there has not been a consistent decrease in fixation failure with the use of cement [23, 24]. Additionally, the use of cement can cause thermal damage to the lesion area, leading to the death of tumor cells [25]. In the study by Kobryn et al., when cemented and non‐cemented intramedullary nails were compared, no difference was found in survival and functional scores, but greater blood loss was observed in the cemented group. The authors suggested that this might be related to selection bias and noted that they used cement more frequently in actual fractures compared to impending fractures [8]. In the study by Laitinen et al., it was observed that cemented nailing provided better pain relief, reduced analgesic usage, and improved functional restoration [4]. A significant risk of cementing is the possibility of it extruding from the fracture site, leading to irritation or thermal damage to surrounding tissues. Particularly, vascular and nerve injuries are among the most serious complications. In our study, no neurovascular problems developed in any of the patients who underwent cementing. Furthermore, no differences were observed between cemented and non‐cemented nailing regarding survival and functional outcomes. Although the use of cement was left to the surgeon's intraoperative preference in this study, the absence of differences in terms of type of cancer and actual‐impending fracture type can be interpreted as a way to avoid bias.

Intramedullary nails are minimally invasive surgeries that allow for small incisions. As a result, the operation time may be shorter, smaller incisions enable earlier radiotherapy and chemotherapy, and early rehabilitation can be facilitated [1, 15, 16, 23]. These nails can provide stability for a long segment due to their intramedullary application. However, intramedullary nails also have potential disadvantages, including tumor spread, inadequate reduction of tumor burden, neurovascular damage, rotator cuff injury, shoulder stiffness, fat embolism, and fixation loss [7, 11, 26]. Rotator cuff injury and shoulder stiffness are significant problems after nail application [1]. Good repair after sharp rotator cuff incisions can help minimize these problems. Embolism, as a complication, is also common with intramedullary nail fixation. Reaming, nail insertion, and cement application can increase intramedullary pressure, potentially leading to cardiopulmonary events. In the treatment of pathological humeral fractures, it is essential to evaluate the risk–benefit ratio carefully. For patients with poor general health who are unsuitable for surgery, non‐surgical approaches should also be considered. Multidisciplinary assessment of life expectancy and functional status can support the decision‐making process for conservative treatment. Nonetheless, due to the limited healing capacity of these fractures, intramedullary nailing plays a key role in providing pain relief and enabling early functional recovery in patients who can tolerate surgery [7, 27, 28]. In this study, five patients died within 2 weeks postoperatively, with one death occurring during intraoperative cement application. Although this outcome may suggest that our surgical indications were relatively broad, we agree with previous reports that severe pain alone can be a sufficient reason to perform intramedullary nailing, given its minimally invasive nature [4, 29]. In one of the patients in this study, fixation loss occurred after the rapid progression of the tumor lesion, and endoprosthetic surgery was performed for the lesion. Additionally, one patient experienced temporary radial neuropraxia, four patients had prolonged wound drainage, and in eight patients who received cement, there were soft tissue extrusions of no clinical significance. No other major or minor complications were observed. Considering the general condition of patients with metastatic bone disease and their susceptibility to complications, we believe that intramedullary nailing will continue to be a feasible option based on the survival and functional outcomes obtained in this study.

### 4.3 Limitations and Strengths

This study has significant limitations, the most important of which are the retrospective design and the sample size. Although similar studies in the literature also observed a comparable number of participants to our study [5, 8, 9, 10, 17, 18, 19], the sample size in our study remains small. In studies with larger sample sizes, more detailed subgroup analyses and more insightful conclusions are likely to be drawn. Although many factors were compared in this study, no comparison group was included regarding surgical treatment. Another limitation is the short follow‐up period, which is influenced by the low survival rate of patients with pathological humerus fractures. Despite these limitations, the study has several notable strengths. It provides a comprehensive evaluation of both functional outcomes and mortality, offering a holistic view of patient condition and the impact of treatment. Moreover, by examining multiple factors related to both function and survival, the study allows for a more detailed interpretation of the outcomes. Finally, the use of multivariate Cox regression analysis strengthens the findings related to mortality.

## 5 Conclusion

This study investigated the factors influencing survival and functional outcomes in metastatic humeral fractures. Accordingly, the presence of cancer type and visceral metastasis was found to have a negative effect on functional outcomes. In patients younger than 65 years, functional scores were superior compared to older patients. It was observed that actual fractures had better pain relief compared to impending fractures, and shaft fractures had better pain relief compared to proximal fractures. Patients who did not have a cancer diagnosis prior to the fracture had better functional outcomes compared to those with a prior diagnosis. Furthermore, while the presence of rapid cancer type and visceral metastasis significantly affects the mortality of these patients, no other factors were observed to independently impact mortality. Although there is an intraoperative and postoperative mortality risk associated with the use of cement, no differences were observed between patients who used cement and those who did not in terms of mortality and functional outcomes.

## Author Contributions

**Ebubekir Eravsar:** conceptualization, methodology, formal analysis, writing – original draft, writing – review and editing, project administration. **Ali Gulec:** validation, investigation, methodology, writing – original draft, formal analysis. **Sadettin Ciftci:** data curation, investigation, formal analysis, writing – original draft. **Numan Mercan:** data curation, investigation, writing – original draft, formal analysis, methodology. **Selim Safali:** data curation, investigation, formal analysis, writing – original draft. **Bahattin Kerem Aydin:** supervision, writing – original draft, writing – review and editing.

## Ethics Statement

Ethical approval was obtained from the Selcuk University Local Ethics Committee. Date: 03.12.2024, Number: 2024/604.

## Consent

The authors have nothing to report.

## Conflicts of Interest

The authors declare no conflicts of interest.

## Data Availability

The datasets generated and/or analyzed during the current study are available from the corresponding author on reasonable request.
